# Use of an Ingestible, Sensor-Based Digital Adherence System to Strengthen the Therapeutic Relationship in Serious Mental Illness

**DOI:** 10.2196/39047

**Published:** 2022-12-02

**Authors:** Anabel G Richey, Ildiko Kovacs, Sara Browne

**Affiliations:** 1 Department of Psychiatry Oxford University Oxford United Kingdom; 2 Department of Medicine University of California, San Diego La jolla, CA United States; 3 Department of Psychiatry University of California San Diego, CA United States

**Keywords:** patient-physician relationship, ingestible sensor, mental health, serious mental illness, antipsychotic, medication adherence, digital adherence, therapy, digital intervention, digital mental health

## Abstract

Serious mental illness is a chronic condition that requires long-term pharmacological treatment. Adherence to oral antipsychotic medication has specific nuances that affects patients and physicians alike. For patients with serious mental illness, nonadherence increases their risk of hospitalization and relapse. Nonadherence is a formidable barrier for physicians in accurately assessing medication efficacy and helping patients achieve their fullest potential. A digital adherence system approved by the Food and Drug Administration can provide near–real time aripiprazole ingestion information. The system records ingestions through an embedded ingestible sensor in oral aripiprazole, which sends a transient local signal to a patch worn on the patient’s torso that is then stored on a paired smartphone app. With patient permission, these data can be viewed remotely by their physician, along with a patient’s mood, activity, and time spent resting. Such data are able to do the following: reveal broad patterns of medication adherence behavior to the patient as well as their physician; help physicians and patients understand and create more realistic expectations for adherence; promote discussion of treatment options; and minimize therapeutic appointment time devoted to determining actual adherence, thereby maximizing the time available to address each patient’s distinctive reasons for their adherence pattern. Crucially, extra time created during appointments can be used to strengthen the therapeutic relationship, which may translate into both improvements in adherence and patient attitude toward their medication. Future investigations are needed to examine how this technology impacts the development of training and best practice guidelines for its use. Otherwise, the potential benefits of this technology may be lost, or worse, inadequate and inappropriate use may harm the therapeutic relationship.

## Introduction

### Overview

The art has three factors, the disease, the patient, the physician. The physician is the servant of the art. The patient must cooperate with the physician in combatting the disease.

Hippocrates

Heralded as the father of western medicine, Hippocrates’ contributions to medicine remain foundational to current perceptions of disease and the body [1]. He used multiple strategies to treat ailments, including herbal remedies in a manner similar to modern prescribed pharmaceuticals [2,3]. Hippocrates’ understood that the medicinal art form requires a compassionate, holistic, yet highly individualized series of investigations into how the patient and disease intertwine. He understood there must be trust and honesty between both parties, and a doctor’s help can only go as far as patients are willing to cooperate. Every patient has the power to render any oral medication 100% ineffective, no matter how much money, time, or human resources the pharmaceutical industry infuses into its creation; a drug is only effective if it is taken [4].

Medication adherence is especially important in cases of serious mental illness (SMI), a term referring to bipolar disorder, schizoaffective disorder, and schizophrenia [5]. Reported adherence rates to antipsychotics vary, ranging from 24% to 90%, with a mean of 58% [6]**.** Nonadherent patients with schizophrenia have 55% higher odds of being admitted to hospital; adequate adherence is imperative to ensure remission and prevent SMI relapse [7].

The first ingestible-sensor–based digital adherence system (iDAS) to follow and support oral medication adherence was approved by the Food and Drug Administration in 2014 [8]. The major advancement associated with this medical device is capture of real-time data on actual drug ingestion along with capture of simultaneous physiological data [9]. Specific approval of the ingestible sensor system with the atypical antipsychotic aripiprazole was obtained in 2017 [10,11]. Aripiprazole, originally approved for use in schizophrenia [12], is also currently approved for use as an adjunct or monotherapy in bipolar I mania [13-17] and major depressive disorder [18]. Approval of digital aripiprazole included the use of an app tailored specifically for patients with serious mental illness under the name Abilify Mycite System [19].

This viewpoint discusses how iDAS adherence data may be used to benefit psychiatric consultations and positively impact the physician-patient relationship. We focus on how iDAS may be used to serve and strengthen the therapeutic relationship (TR), foundational to any beneficial outcome of any technology application [20]. Could iDAS represent a new era of physician-patient interactions, a technological turning point that Hippocrates could not have foreseen?

For the sake of clarity, at the outset, our discussion assumes patients give informed consent before and throughout their use of iDAS. We focus on how iDAS may be used to serve and strengthen the TR, which is foundational to any beneficial outcome of any technological application [20]. The ethics of informed consent is of critical importance when using this technology (see analysis and discussion by Beriain and Gonzalez [21] and Torous and Roberts [20]). As the cornerstone of any TR is trust, and a large portion of such trust comes from continued consent over time, we subsequently discuss dynamic consent during iDAS use.

### Background

**Figure 1:**
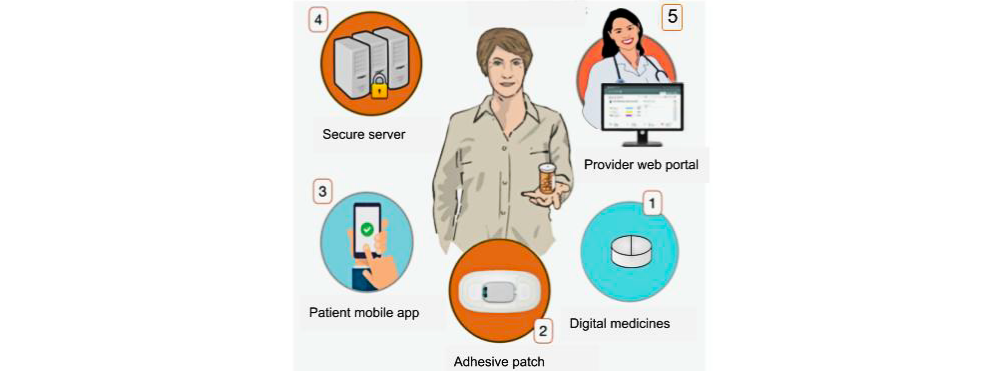
Depiction of the components of aripiprazole ingestible-sensor–based digital adherence system (iDAS). (1) Aripiprazole with an embedded ingestible sensor, a patch, and paired smartphone with an installed iDAS app. (2) When ingested and in contact with gastric fluids, the sensor sends an electrochemical signal unique to aripiprazole that is recognized and stored by a patch worn on the user’s torso. (3) The patch then communicates via Bluetooth to the patient’s paired smartphone with an installed app that stores the time and date of successful medication ingestion. (4-5) The patient allows the prescribing provider to view ingestion data via a website that is accessed through a secure server.

A Food and Drug Administration–approved iDAS, comprising the ingestible sensor and a generic app [8], has been used with multiple oral medications [22-24]. The iDAS for aripiprazole, developed in collaboration with Otsuka America Pharmaceutical, Inc [25], was approved with an app developed specifically for the use of patients with SMI and released under the name Abilify Mycite System [26]. The iDAS for aripiprazole has 3 components, which are aripiprazole with embedded ingestible sensor, a patch, and a paired smartphone with installed iDAS app (Figure 1). When ingested and in contact with gastric fluids, the sensor sends an electrochemical signal unique to aripiprazole that is recognized and stored by a patch worn on the user’s torso. The patch then communicates via Bluetooth to the patient’s paired smartphone with installed app, which stores the time and date of successful medication ingestion [27]. Over 90% of pill ingestions are detected by the patch in 3 minutes, while the patch can take up to 2 hours to sync ingestion data with the app [28,29]. The app has several features, including tracking mood, rest, and activity.

**Figure 2:**
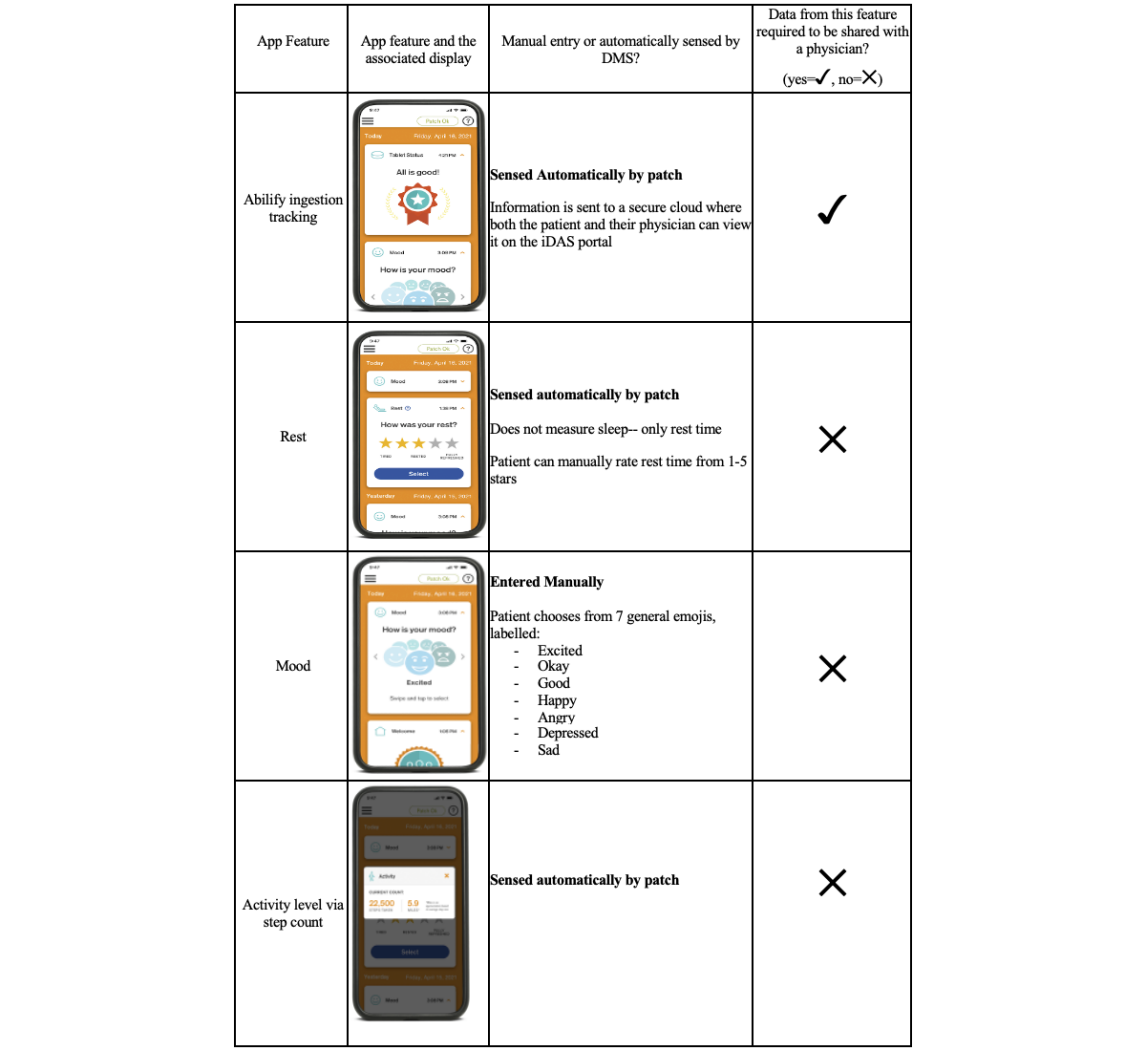
Features of the aripiprazole iDAS app. All pill ingestion information automatically enters the app and must be shared with a health care provider with patient’s permission. Other features, including mood, rest, and activity, do not have to be shared with a physician. All features of the app can be shared with 5 other people including friends or family, or another physician. iDAS: ingestible-sensor–based digital adherence system. (Images adapted and used with permission from MyCite Menu Overview, 2021, Otsuka America Pharmaceutical, Inc).

Figure 2 shows the displays of each feature in addition to noting if the feature’s data are manually entered. The patient may give permission for their physician (and other trusted persons) to access a web-based iDAS portal where their medication adherence data can be view remotely. The app currently makes clear attempts to gather a holistic data set, including information on mood and activity from the patient. It uses friendly interface displays such as “You’re all good!” once a pill is ingested. The app has a *casual* appearance and does not, for example, display, “You’re all good! Aripiprazole ingested: 11:24 AM. Ingestion data sent to Dr. Argus Panoptes at 12:24 AM.” The *feel-good* design of iDAS chooses not to highlight physician monitoring, for better or for worse.

The type of data collection by iDAS contributes to the underappreciated “art of observation of the individual in its entirety” [30], providing objective inside data about a patient’s habits, thus falling under the umbrella of devices that contribute to digital phenotyping, as defined by Torous et al [31]. Several other tools have been developed to integrate digital phenotyping data into patient care, including The SilverCloud platform using digital phenotyping data to delineate different subtypes of internet-based cognitive behavioral therapy [32] and the MindLAMP platform, aimed at preventing relapse in people with schizophrenia spectrum disorders [33,34].

Abilify iDAS is intended to augment treatment as usual by a physician, a model incorporated into the MindLAMP [34] and Horyzons platforms [35]. Abilify iDAS and these platforms have consistently demonstrated feasibility of use and efficacy with treatment as usual in psychiatric patients with SMI [34,36-41]. These reports substantiate the proposal that Abilify iDAS could support the TR. By and large, more data on patients are not inherently *beneficial* nor *harmful*; rather, there are needs for careful consideration of what information the iDAS can provide and how this may be used to build trust and mutual understanding in psychiatric consultations.

## What Near–Real Time Adherence Data May Bring to Psychiatric Consultations

### Confirmation of a Patient’s Adherence

The iDAS technology allows for an accurate summary of medication adherence available for medical visits. Through no fault of their own, patients may demonstrate recall bias at appointments by erroneously reporting medication adherence or experience [42]. The physician and patient can look back at the adherence data together and discuss how medication adherence is going.

### Inform Dosage Adjustment

The features of the iDAS may inform physicians tailoring the aripiprazole dosage. Two common aripiprazole side effects include restlessness and akathisia as well as somnolence [43]. iDAS ingestion data, in conjunction with rest and activity data, may be helpful in gauging a patient’s experience with these side effects. In theory, a doctor could adjust the aripiprazole dose in a patient who complains of being unable to sit still, frequently rates their rest at 1 star, and has relatively good medication adherence. It is important to note, however, that Abilify iDAS is not licensed to inform the modification of aripiprazole dosage [44]. Nonetheless, real-time rest information in a patient complaining of somnolence would be difficult to ignore. In situations where the patient’s app data and verbal recollection concur, a physician may feel more assured in changing a patient’s dose.

### Temporal Tracking of SMI Relapse Relative to Medication Adherence

Another benefit of the aripiprazole iDAS is the potential to track whether a symptomatic relapse was caused by nonadherence or a medication’s lack of efficacy. As Weiden [45] describes, relapse caused by inefficacy of a medication may begin prior to nonadherence, and without clear data, relapses can be misattributed to behavioral nonadherence. To illustrate this, a clinician may assume that their patient ceased medication and relapsed, leading them to encourage their patient to resume their medication. However, using the aripiprazole iDAS, a clinician could review ingestion data and build a more accurate timeline of medication cessation in relation to their patient’s relapse. If the physician concludes that the relapse began before medication cessation, the physician can adjust the treatment regimen. Similarly, physicians can also see when, despite maintaining good adherence throughout, a patient relapsed and clearly requires medication dosage or regimen changes.

### Improve Patient’s and Physician’s Understanding Patterns of Adherence

The duration and reliability of iDAS data allow patients and physicians to isolate trends in adherence behavior. Much of the discussion of the commercial Abilify Mycite product focuses on its advantage to physicians, who are required to prescribe it. Crucially, aripiprazole iDAS data may also aid a patient in learning about themselves. Seemingly random instances of nonadherence may be associated with broader patterns. These patterns may be illuminated by simultaneously considering the rest, activity, and mood recordings with ingestion data over time. Browne et al [9] produced visualizations coupling iDAS physiological data with adherence data in patients with diabetes, which allowed easily accessible interpretation of individual behavior patterns over time. iDAS data can provide the opportunity for patients to use ingestion data as a lens to reveal patterns in their own life, rhetorically asking, “What does nonadherence tell me about my own life?” Physicians may use adherence data in an opposite fashion: life patterns can be used as a lens to reveal adherence data. A physician may rhetorically ask, “What does a patient’s life tell me about their nonadherence?”

Thus, the iDAS system can be used as a recordkeeper to help understand patterns of adherence in patients, in ways that directly benefit the psychiatric consultation. After all, Hippocrates states that physicians are servants “to the art of medicine” rather than patients being servants to the physician. All art forms involve flexibility coupled with creativity, but this art form, as every physician knows, requires the most precious commodity of all in medical practice, which is *time*.

## Time Opened up During Appointments May Support the Therapeutic Relationship

Easily accessible and highly accurate adherence data provided by iDAS shorten time spent on adherence *detective work* on the part of the physician during consultations. This opens up appointment time, which may be used to start healthy conversations around adherence patterns. In a study of conversations surrounding antipsychotic nonadherence during psychiatric consultations, patients often tried to minimize the risk of a “disciplinary” reply from their physician when disclosing nonadherence [46]. Patients are frequently ashamed of their noncompliance, especially when they are attached to their treating physician. To illustrate this, 1 patient began the disclosure of nonadherence by saying “My mood’s been fine but you’re not gonna be very happy [to know] …” [46]. This study of nonadherence disclosures highlights how difficult they can be for the patient. The authors compare patients’ methods of nonadherence disclosure to methods used to deliver *bad news*. Patients should not worry or feel embarrassed by medication nonadherence as there can be many reasons for it. While the physician needs to spend time, interpreting patterns provided by iDAS, this can be conducted with the patient, spending more time exploring reasons for their behavior patterns.

## Closing the Loop: Strengthening the Therapeutic Relationship as a Means to Antipsychotic Treatment Adherence

The value of adherence data cannot be overstated, even in situations where patients seem to be making little progress in becoming adherent to their medication regime. How can iDAS be valuable when the patient is firmly nonadherent, despite a kind and honest effort from their physician? In fact, these situations of firm nonadherence may create tension between the physician and patient, creating the sense that both parties are at a *standstill*. Nevertheless, true medication adherence cannot exist without a patient’s notion of trust and confidence in their physician. The availability of iDAS data means time—which would otherwise have been spent trying to investigate whether a patient is adherent to their regime at all—can be spent to improve this trust during consultations.

In their book on therapeutic alliance, Leslie Greenberg and Adam Horvath explicitly state that the core of therapeutic alliance relies on a sense of collaboration [47]. One cannot create a genuine collaboration with someone they do not trust. Therapeutic alliance is contingent on factors beyond agreement of treatment-related goals, with one of the most important factors being the personal bond between patient and physician [47,48]. Forming such a bond takes time. Extra appointment time can facilitate this bond, particularly when there may not be agreement between a patient and physician.

The bond contributing to therapeutic alliance requires mutual trust and regard [49]. Extra appointment time facilitated by iDAS use may be spent strengthening the therapeutic alliance by openly and nonjudgmentally discussing possible reasons for nonadherence, trying to find the specific reasons why the individual patient is not taking the medication as prescribed [50]. Most patients are not aware of the multiple layers of attitudinal and behavioral factors leading to therapeutic adherence; when it is discussed in a cooperative, nonthreatening, and constructive way, patients are usually very interested in understanding their own behaviors [51]. Physicians need to provide encouragement and positive feedback for patients’ effort [52].

Patients with schizophrenia who established a good therapeutic alliance during the first 6 months of treatment had a significantly increased likelihood of adhering to medication regimes [53]. This initial alliance was also significantly associated with improved treatment outcomes using less medication when compared to patients without good therapeutic alliance [54]. Subsequent studies on TR and antipsychotic adherence in patients with schizophrenia demonstrated that TR is associated with both adherence and attitude toward treatment with antipsychotics [54]. The use of the Helping Alliance Scale [54,55] to measure TR strength in clinicians and their patients (higher scores signify a better TR) showed each unit increase in the clinician’s TR rating was accompanied by a 65.9% increase in the odds ratio of their patient having good medication adherence. For each patient-rated TR unit increase, there was a 20.8% increase in the odds ratio of good medication adherence [54]. Consequently, extra time spent strengthening the physician-patient bond could lead to better adherence in patients taking antipsychotics.

### Dynamic Consent With Third Party Availability During iDAS Use May Minimize Risks to the Therapeutic Relationship

The bond that forms the cornerstone of the TR is based on mutual trust between physician and patient, which not only builds over time, but may also change over time. To support this bond over time iDAS used with dynamic consent may increase trust. Prictor et al [56] defined dynamic consent as “an approach to consent that enables people, through an interactive digital interface, to make granular decisions about their ongoing participation.” The iDAS MyCite app contains inclusion of a digital interface allowing patients at any time to withdraw consent to all or certain types of data sharing with some (or all) of previously approved persons, including their physician [57]. Of course, it would be ideal if a patient felt comfortable telling their doctor that they no longer want certain portions of their data shared with their health care team. However, some patients may prefer to withdraw consent on a digital platform by clicking a “stop sharing data” button. It may also be important to consider having access to a third party, such as a nurse or a *consent representative*, to allow the patient to discuss their ongoing iDAS consent and relationship with the doctor. This would allow open conversations about withdrawing consent to be made outside of the doctor-patient space and create a space of open conversations about data sharing without a *direct* disruption in the doctor-patient relationship. The availability of a third party, such as a nurse, can directly address questions of “Who do you want to allow to see your data?”, “What data are they allowed to see?” and “How do you want these data to be used?” This would allow a patient to discuss their patient-physician relationship, patient-data relationship, and physician-data relationship with someone in health care who is not their doctor. The third-party patient advocate role would mitigate patient’s concerns about withdrawing consent and would also allow a patient to feel that their feelings and concerns are their health care team’s *priority.* Dynamic consent, in addition to the availability of a third party, facilitates methods for key patient data control proposed by Vayena and Blasimme [58]—control over who accesses one’s health care data and how those data are used. Placing the patient at the center of the consent process over time would likely optimize patient autonomy and physician-patient confidence that iDAS was supporting their relationship.

### Conclusion

Assuming a patient agrees to use it over time, the aripiprazole iDAS can provide information on an individual patient’s adherence, which may help to inform dosage and trace SMI relapse relative to medication adherence. iDAS data can be an *icebreaker* in conversations surrounding medication adherence or how patients feel about their current medication regime. Through iDAS use, patients may learn more about their relationship with adherence, and physicians may gain a more accurate perspective on individual patients, improving both the patient and physician’s understanding of adherence patterns. Ultimately, this tool only reports behavioral data, which does not change a patient’s attitude toward antipsychotics. It is up to the physician to practice their art in a way that compassionately understands and encourages patients to reach their full potential. This can only be done through time spent developing a strong and healthy therapeutic relationship, which itself has repeatedly been shown to influence patient treatment adherence. As data accumulate from patients and their physicians choosing to use the aripiprazole iDAS, a more complete picture will emerge of opportunities to tailor and provide targeted use needed for different SMI diagnoses and individual patient characteristics, such as gender. It is critical, however, that future investigations examine how this technology impacts the physician-patient relationship to develop training and provide best-practice guidelines on how to use it to strengthen the therapeutic relationship. Otherwise, the potential benefits of this technological advancement may be lost, or worse still, inadequate and inappropriate use may result in harm to the therapeutic relationship.

## Abbreviations

iDAS: ingestible-sensor–based digital adherence system
SMI: serious mental illness
TR: therapeutic relationship
